# Correction: HDX-MS reveals structural determinants for RORγ hyperactivation by synthetic agonists

**DOI:** 10.7554/eLife.52847

**Published:** 2019-10-18

**Authors:** Timothy S Strutzenberg, Ruben D Garcia-Ordonez, Scott J Novick, HaJeung Park, Mi Ra Chang, Christelle Doebellin, Yuanjun He, Rémi Patouret, Theodore M Kamenecka, Patrick R Griffin

Strutzenberg TS, Garcia-Ordonez RD, Novick SJ, Park H, Chang MR, Doebellin C, He Y, Patouret R, Kamenecka TM, Griffin PR. 2019. HDX-MS reveals structural determinants for RORγ hyperactivation by synthetic agonists. *eLife* **8**:e47172. doi: 10.7554/eLife.47172.

Published 7, June 2019

The phenylalanine residues in Figure 1A are labelled with incorrect numbers. The original labels of F377 and F378 were changed to F378 and F388 to match the structure numbering. This residue numbering error does not occur in the text and does not change any of the conclusions of the paper.

The article has been corrected accordingly.

